# Rationale for a Multimodality Strategy to Enhance the Efficacy of Dendritic Cell-Based Cancer Immunotherapy

**DOI:** 10.3389/fimmu.2015.00271

**Published:** 2015-06-02

**Authors:** Jashodeep Datta, Erik Berk, Jessica A. Cintolo, Shuwen Xu, Robert E. Roses, Brian J. Czerniecki

**Affiliations:** ^1^Division of Endocrine and Oncologic Surgery, Department of Surgery, University of Pennsylvania Perelman School of Medicine, Philadelphia, PA, USA; ^2^Rena Rowen Breast Center, Hospital of the University of Pennsylvania, Philadelphia, PA, USA

**Keywords:** dendritic cell, immunotherapy, multimodality, adoptive cell therapy, targeted therapy, checkpoint inhibitor, chemotherapy, radiotherapy

## Abstract

Dendritic cells (DC), master antigen-presenting cells that orchestrate interactions between the adaptive and innate immune arms, are increasingly utilized in cancer immunotherapy. Despite remarkable progress in our understanding of DC immunobiology, as well as several encouraging clinical applications – such as DC-based sipuleucel-T for metastatic castration-resistant prostate cancer – clinically effective DC-based immunotherapy as monotherapy for a majority of tumors remains a distant goal. The complex interplay between diverse molecular and immune processes that govern resistance to DC-based vaccination compels a multimodality approach, encompassing a growing arsenal of antitumor agents which target these distinct processes and synergistically enhance DC function. These include antibody-based targeted molecular therapies, immune checkpoint inhibitors, therapies that inhibit immunosuppressive cellular elements, conventional cytotoxic modalities, and immune potentiating adjuvants. It is likely that in the emerging era of “precision” cancer therapeutics, tangible clinical benefits will only be realized with a multifaceted – and personalized – approach combining DC-based vaccination with adjunctive strategies.

## Introduction

Dendritic cells (DCs) function at the interface of the innate and adaptive immune systems, making them uniquely suited for cancer immunotherapy. As sentinel members of the innate immune arm, DCs elaborate protective cytokines (i.e., IL-6, IL-12) in response to “danger” signals (1). As master antigen-presenting cells (APC), DCs capture, process, and present antigens in the context of major histocompatibility (MHC) molecules to naïve T-cells at lymphoid organs, thereby inducing adaptive CD4^+^ and CD8^+^ T-cell-mediated immune responses (2, 3); indeed, DCs’ potency for inducing T-cell proliferation is 10–100 times that of B-cells or monocytes (4).

Unique properties make DCs particularly attractive vehicles for immunotherapy. These include their ability to cross-present (i.e., re-route exogenous antigens typically presented on MHC class II molecules into pathways for class I presentation) (5), induce natural killer (NK) or NK T-cell responses (6, 7), and potentiate antitumor humoral responses (8). More importantly, plasticity of DC lineage and the ability to direct DC activation with external signals [e.g., Toll-like receptor (TLR) agonists], which polarize ensuing T-cell responses, can be harnessed for therapeutic application in DC-based approaches (9).

Following the initial promise of DC-based vaccination attempts in lymphoma and melanoma patients (10, 11), autologous DCs have been employed in immunotherapy for several tumor types, including melanoma, prostate cancer, renal cell carcinoma (RCC), and glioblastoma with varying success. A majority of these trials indicate that DC-based immunotherapy, while tolerable and strongly immunogenic, fails to achieve meaningful objective response rates (12). These data, along with the remarkable diversity of cytokine activation regimens, DC maturation states, and antigen loading strategies employed in DC vaccine design (9), reflect an evolving – but incomplete – understanding of optimal DC immunobiology. As such, despite recent FDA approval of sipuleucel-T – blood DCs pulsed with prostatic acid phosphatase-GM-CSF fusion protein – for metastatic castration-resistant prostate cancer (13), clinically effective DC immunotherapy as *monotherapy* for a majority of solid tumors remains a distant goal.

**Figure 1:**
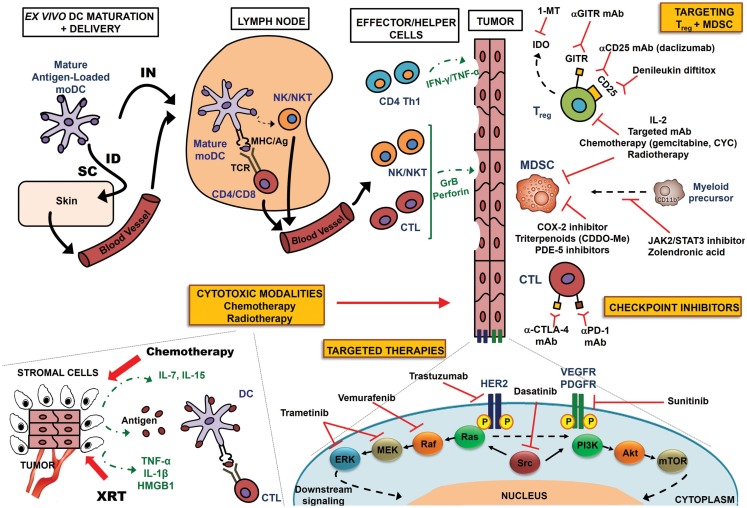
**Multimodality approach to optimize DC-based immunotherapy**. Antigen-specific T-cell responses can be induced by traditional *ex vivo*-manipulated DCs or DC receptor targeting *in vivo* (not shown in this schematic). In *ex vivo* manipulation, monocyte precursors are sequentially matured with proinflammatory cytokines, loaded with antigen, and injected either IN or ID/SC. Lymph nodes serve as sites of T-cell co-stimulation, whereby DCs present antigen to T-cells in the context of MHC Class I/II molecules, triggering antigen-specific CD4^+^ Th1 cells or CD8^+^ CTLs. DCs also have the unique ability to induce other immune effectors, such as NK and NK T-cells. These effector and helper populations migrate to the tumor bed, where they directly attack tumor cells via GrB/perforin (CTL or NK/NKT cells), or elaborate cytokines (e.g., Th1 cytokines IFN-γ and TNF-α) to mediate apoptosis. Multimodality enhancement of DC-based immunotherapy may be achieved by one or more of the following mechanisms: (a) conventional cytotoxic modalities: lymphodepleting chemotherapy regimens generate an immune recovery cytokine environment via elaboration of IL-7, IL-15, etc.; irradiation (XRT) of tumor cells induces release of tumor-associated antigens, pro-inflammatory cytokines (IL-1β, TNF-α), or endogenous TLR agonists (HMGB-1), activating DCs to prime antigen-specific CTL responses; antigens may also be presented by stromal cells for destruction by CTLs; (b) mAb-based targeted molecular therapies – targets of translatable promise are shown, including HER2 (trastuzumab), VEGFR/PDGFR (sunitinib), BRAF (vemurafenib), MEK/ERK (trametinib), and Src (dasatinib); such blockade abrogates downstream nuclear signaling and inhibits proliferation; (c) preventing activated CTL “exhaustion” with checkpoint inhibitors targeting CTLA-4 and PD-1 – immunostimulatory therapies aimed at recovering T-cell cytotoxicity; (d) muting tumor-elaborated T_reg_ and MDSCs. A variety of agents, including IL-2, targeted mAbs, chemotherapy regimens, and radiotherapy can dually inhibit T_reg_ and MDSC function. COX-2 inhibitors, PDE-5 inhibitors, and triterpenoids can selectively target MDSCs, while JAK2/STAT3 inhibitors and zolendronic acid prevent myeloid differentiation to a suppressor phenotype. Anti-CD25 mAbs and denileukin diftitox (CD25) or anti-GITR mAb (GITR) target receptors specific to T_reg_, whereas 1-MT inhibits T_reg_-elaborated IDO.

There is emerging evidence that the maximal benefit of DC-based immunotherapy may be achieved in *combination* with other antitumor therapies that augment DC function (Table 1; Figure 1). In this review, we explore the biologic rationale for such multimodality approaches to optimize the impact of current DC-based cancer immunotherapy.

**Table 1:** **Multimodality strategy to enhance the efficacy of dendritic cell-based vaccination**.

Strategy	Agent/technique utilized		Proposed advantage(s)	Clinical trial(s) completed/underway, if applicable
Adoptive cell therapy (ACT)	Autologous T-cells/TIL		Fewer adverse effects, circumvent need for pre-conditioning with chemotherapy, IL-2, etc.	**Melanoma** (NCT01946373, NCT00338377, NCT00910650, NCT00313508, NCT00961844, NCT01339663); **Brain** (NCT00693095, NCT01759810); **Breast** (NCT01782274); **Lung** (NCT01782287, NCT00776295)
	Genetically engineered TCR or CAR T-cells		Synergistically enhance antigen targeting and DC function	**Melanoma** (NCT00910650); **Solid** (NCT00704938, NCT01697527)

Targeted therapies	Sunitinib	}	Inhibits MDSC, depletes CTLA-4/PD-1	**Renal** (NCT01582672, NCT00678119)
	Dasatinib			**Melanoma** (NCT01876212)
	Trastuzumab		Potentiate CTLs, enhance ADCC	**Breast** (NCT00088985, NCT00266110, NCT02336984)
	Vemurafenib		Potentiate DC function	–

Targeting immune checkpoint pathways	Anti-CTLA4		Inhibit CTLA-4:B7	**Melanoma** (NCT00090896)
	Anti-PD-1		Impair PD-1:CTL interaction	**Renal** (NCT01441765); **Prostate** (NCT01420965); **Hematological** (NCT01096602, NCT01067287)

Muting immunosuppressive cellular elements	Anti-CD25 (basiliximab, daclizumab) mAb		Deplete T_reg_	**Brain** (NCT00626483); **Melanoma** (NCT00847106); **Ovarian** (NCT01132014)
	Denileukin diftitox		Target CD25, deplete T_reg_	**Melanoma** (NCT00056134); **Ovarian** (NCT00703105); **Solid** (NCT00128622)
	1-methyl-d-tryptophan		Inhibits indoleamine-2,3-dioxygenase	**Breast** (NCT01042535)
	All-trans retinoic acid		MDSC differentiation into non-suppressive cells	**Lung** (NCT00617409)
	COX-2 inhibitors (celecoxib, meloxicam)		Inhibit CCL2, upregulate CXCL10	**Melanoma** (NCT00197912); **Head and Neck** (NCT00589186); **Brain** (NCT01759810); **Lung** (NCT00442754, NCT01782287); **Breast** (NCT01782274)
	Anti-VEGF		Inhibit MDSC	**Renal** (NCT00913913); **Prostate** (NCT00027599); **Ovarian** (NCT00683241 NCT01132014)

Chemotherapy	Cyclophosphamide ± fludarabine		Lymphodepleting, reboots immune system	**Solid** (NCT01697527); **Brain** (NCT00323115, NCT02010606); **Melanoma** (NCT00338377, NCT00910650, NCT01946373, NCT00313508, NCT00704938); **Renal** (NCT00704938, NCT00093522)
	Metronomically dosed cyclophosphamide		Depletes T_reg_/MDSC, potentiates Th1	**Head and Neck** (NCT01149902); **Lung** (NCT01159288); **Melanoma** (NCT00197912, NCT00683670, NCT00722098, NCT00978913, NCT00313235, NCT01339663; NCT00610389), **Mesothelioma** (NCT01241682); **Ovarian** (NCT00683241, NCT00478452); **Prostate** (NCT01339663); **Renal** (NCT00610389)
	Gemcitabine		Improves cross-presentation, T_eff_ infiltration	**Pancreatic** (NCT00547144); **Sarcoma** (NCT01803152)
	Temozolomide		Immune recovery cytokine environment	**Brain** (NCT00323115, NCT01213407, NCT01567202, NCT00639639); **Melanoma** (NCT00961844)

Radiotherapy	Radiotherapy		Enhances tumor immunogenicity, releases TLR agonists, targets stroma, abscopal effect	**Brain** (NCT00323115, NCT01213407, NCT01567202); **Breast** (NCT00082641); **Esophageal** (NCT01691625); **Melanoma** (NCT00278018); **Pancreatic** (NCT00547144, NCT00843830); **Sarcoma** (NCT00365872, NCT01347034)

Cytokines and TLR agonists	IL-2		Protect CTL effectors from tumor-mediated dysfunction	**Brain** (NCT01235845); **Breast** (NCT00197925); **Colorectal** (NCT00176761, NCT0001959); **Lung** (NCT00442754); **Melanoma** (NCT00197912, NCT00338377, NCT00910650, NCT00279058, NCT00006113, NCT00004025, NCT01339663, NCT00003229, NCT00019214, NCT00704938); **Renal** (NCT00197860, NCT00913913, NCT00085436, NCT00704938); **Sarcoma** (NCT00001566); **Lymphoma** (NCT00006434)
	Poly-I:C or derivatives (TLR3)		DC activation, T_eff_ infiltration	**Melanoma** (NCT00278018, NCT00610389); **Renal** (NCT00913913, NCT00085436, NCT00610389)
	IFN-α		Induce apoptosis of tumor	**Myeloma** (NCT00616720)
	IFN-γ		Cytotoxic, polarize Th1	**Pediatric Solid Tumors** (NCT00923351)
	IL-7		Maintenance of DCs	**Breast** (NCT00622401)
	IL-12		Polarize Th1, anti-angiogenic	**Brain** (NCT01808820, NCT01792505, NCT01171469); **Lung** (NCT00442754); **Ovarian** (NCT00799110); **Sarcoma** (NCT01803152, NCT01241162, NCT00944580)
	Imiquimod (TLR7)		Induced type 1-IFN by plasmacytoid DCs	**Brain** (NCT01204684, NCT00766753); **Melanoma** (NCT01783431); **Pancreatic** (NCT01677962, NCT01410968); **Solid** (NCT01734564, NCT02151448)
	Resiquimod (TLR7/8)		T_eff_ infiltration, inhibit T_reg_	**Brain** (NCT01204684)
	Thymosin-α-1 (TLR9)		Potentiate CTL responses	**Renal** (NCT00197860)

*Clinical trials utilizing the respective approach are listed, if applicable*.

*TIL, tumor-infiltrating lymphocyte; TCR, T-cell receptor; CAR, chimeric antigen receptor; ADCC, antibody-dependent cellular cytotoxicity; T_eff_, effector T-cells*.

## Improving Efficacy of Existing DC-Based Vaccines

Traditionally, two DC-based vaccination approaches have been widely used: direct targeting of antigens to DC receptors *in vivo*, and *ex vivo*-generated antigen-loaded DCs. Beyond these approaches – reviewed extensively elsewhere (14) – our growing understanding of DC biology highlights potential strategies to improve DC-based vaccine efficacy: (a) exploiting diversity of DC lineage [i.e., plasmacytoid DCs (15), CD141^+^ DCs (16)] to improve antigen cross-presentation and potency of cytotoxic CD8^+^ T-lymphocyte (CTL) responses; (b) silencing of antigen presentation “attenuators” [e.g., inhibition of SOCS1 (17)] to enhance DC function by controlling the tolerogenic state of DCs and magnitude of antigen presentation; (c) synergizing with adoptive cell therapy [e.g., DC vaccine-primed peripheral blood T-cells expanded *ex vivo* with CD3/CD28 co-stimulation (18)]; (d) manipulating *ex vivo* DC maturation conditions to enhance immunogenicity [e.g., utilizing IL-15 to generate Langerhans-type DCs (19), or IFN-γ and lipopolysaccharide (LPS, a TLR4 agonist) to yield type 1-polarized DCs (DC1) (20)]; and (e) modification of co-stimulatory molecule expression to improve DC potency [e.g., mRNA-electroporated DCs encoding CD40L, CD70, and TLR4 (21)].

Three such strategies merit discussion. Adoptive cell therapy (ACT) encompasses infusion of *in vitro*-expanded tumor-infiltrating lymphocytes (TILs) (22–24) or T-cells genetically engineered to harbor T-cell receptors (TCR) – and more recently chimeric antigen receptors (CAR) – specific for tumor-associated antigens (25, 26). While promising, ACT is rarely effective as monotherapy for a majority of tumors; moreover, immune “conditioning” with lymphodepleting chemotherapy (see Section “Cytotoxic Chemotherapy”), total body irradiation, or *in vivo* IL-2 support is needed in order to optimize antitumor efficacy (27). An alternative to these toxic conditioning regimens may be provision of antigen in the form of peripheral DC vaccination, a premise that is supported by several preclinical models (28–31) and early in-human trials (27, 32). Antigen-pulsed DC vaccination may potentiate the proliferation, persistence, and selective migration of transferred T-cells to tumor sites (28). Moreover, the magnitude of the polarized ACT T-cell response may be augmented by DC vaccination via provision of co-stimulatory signals (18). Several trials investigating such combinations are currently underway (Table 1).

While the optimal DC phenotype for cancer immunotherapy remains controversial, it is increasingly recognized that incorporation of IL-12p70-producing DC1 – which subsequently polarize naïve CD4^+^ T-cells toward a IFN-γ and TNF-α-secreting T-helper type 1 (Th1) phenotype (20) – appears advantageous. Our group (33), as well as others (34), employs a streamlined recipe of IFN-γ and LPS to generate high IL-12p70-producing DC1. IL-12p70 – predictive of favorable outcomes in melanoma (35) and glioblastoma (36) patients – promotes NK cell activation (37) and possesses anti-angiogenic properties (38). In our studies, CD8^+^ T-cells could only recognize HLA-A2^pos^ cancer cells if sensitizing DCs secreted IL-12p70 (39). Furthermore, Th1-derived IFN-γ/TNF-α are critically important for tumor rejection in preclinical models (40) and synergistically induce apoptosis of tumor cells *in vitro* (41). Generation of Th1 subsets offers other advantages: Th1-driven CTLs detect class I-tumor antigen complexes with higher affinity than Th2-driven counterparts (42), and are instrumental in B-cell responses by inducing antibody class-switching and IgG production (4).

A potential drawback of DC maturation with IFN-γ/LPS regimens is the narrow temporal window for IL-12p70 secretion – secretion commences around 6 h after IFN-γ/LPS activation; production is maximized – so-called “burst” – around 8–10 h but is virtually exhausted 16–24 h later (20). Vaccination with such exhausted DCs would likely polarize tolerogenic (e.g., Th2) T-cell responses (43), resulting in suboptimal clinical outcomes. Moreover, IFN-γ/LPS activation generates DCs which lack CCR-7 and CXCR-4 chemokine expression, limiting their “trafficking” ability to lymphoid organs (44). To overcome these limitations, our group employs a protocol whereby DCs are: (a) harvested 6 h after LPS activation, prior to IL-12p70 secretory “burst;” and (b) injected intranodally via ultrasound guidance in order to co-localize IL-12p70 “burst” with the anatomic site of T-cell sensitization (20, 45, 46). In general, vaccine design must exploit such pre-programed cytokine secretion schedules in order to optimize *in vivo* DC efficacy.

IFN-γ/LPS-activated DCs are also capable of a second IL-12p70 burst *in vivo* following restimulation in lymph nodes by activated CD4^+^ T-helper (Th) cells via CD40-CD40L interactions (20, 46). Beyond perpetuation of IL-12p70 secretion, CD40 triggering is critical in upregulating co-stimulatory molecule expression (i.e., CD80, CD86) on DCs, promoting cross-priming to exogenous antigens, augmenting CD4^+^ and CD8^+^ T-cell expansion, rescuing CD8^+^ T-cell exhaustion, and mediating resistance of mature DCs to suppression by regulatory T-cells (T_reg_) (47–53). Notably, CD40–CD40L interaction – but not TLR4 signaling via LPS – can restore the capacity for IL-12p70 secretion in IFN-γ/LPS-activated DCs which have exhausted their potential for cytokine secretion (54, 55). Consequently, incorporation of CD40 ligation has emerged as an attractive strategy to enhance DC potency. For instance, autologous DCs electroporated with mRNA encoding CD40L (plus CD70 and TLR4) and fusion protein of an HLA class II-targeting signal (DC-LAMP) and melanoma-associated antigens (TriMixDC-MEL) were immunogenic and generated tumor responses in chemorefractory melanoma (21, 56).

## Targeted Molecular Therapies

The advent of molecular therapies targeting tumor oncogene drivers represents one of the most significant advances in contemporary cancer therapy. Despite encouraging success in many tumors types, however, disease relapse is observed in a sizeable proportion of patients treated with these agents. Novel combinations of targeted therapies with immune interventions, therefore, are conceptually appealing and are being increasingly explored in order to reduce treatment failures (57). A particularly promising candidate is sunitinib, a receptor tyrosine kinase (RTK) inhibitor targeting VEGFR, PDGFR, c-KIT, and Flt-3; in preclinical models, sunitinib decreased tumor microenvironment (TME) accumulation of myeloid-derived suppressor cells (MDSCs), restored Th1/CTL functionality, muted PD-L1 expression on tumor-resident DCs, depleted CTLA-4/PD-1 expression on activated CTLs, and inhibited production of inhibitory IL-10, TGF-β, and FoxP3 from TILs (58, 59). In a phase II clinical trial, administration of sunitinib with DCs co-electroporated with amplified tumor and synthetic CD40L mRNA yielded supportive immune responses and extension of long-term survival in 21 patients with advanced RCC (60).

In preclinical murine models of mutant-BRAF (BRAF^V600E^) melanoma, BRAF^V600E^ inhibitor vemurafenib synergized with Th1 cytokines IFN-γ/TNF-α to induce growth arrest (61). In a separate study, vemurafenib reversed BRAF^V600E^ melanoma-induced DC dysfunction without deleterious effects on DC viability or capacity to prime T-cell responses *in vitro* (62), strengthening its candidacy for combination DC-based immunotherapy.

In a murine B16-OVA melanoma model, combination therapy with dasatinib – a RTK inhibitor targeting BCR-ABL, SRC, c-KIT, and PDGFR – and OVA-pulsed DC1 vaccines decreased TME levels of MDSCs and T_reg_, enhanced TME recruitment of IL12p70-producing DC1, and promoted a profound spreading in the repertoire of tumor-associated antigens recognized by CD8^+^ TILs (63).

We have recently demonstrated that cooperation between DC1-driven Th1 cytokines IFN-γ/TNF-α and HER2/*neu*-targeted antibody trastuzumab is necessary for restoration of MHC class I expression in HER2-overexpressing, but not HER2-low, cancer cells *in vitro*, thereby facilitating recognition and lysis of these cells by DC1-sensitized HER2-specific CD8^+^ T-cells. Activation of EGFR and HER3 signaling abrogated IFN-γ/TNF-α and trastuzumab-induced class I restoration; however, concomitant EGFR/HER3 receptor blockade rescued class I expression and ensuing CD8^+^ T-cell cytotoxicity of HER2/*neu*-expressing cells (64). Therefore, combinations of DC1-directed Th1 immune interventions and multivalent molecular targeting of HER family members may be essential for optimal HER2/*neu*-directed immunotherapy.

Collectively, these data provide strong rationale for DC-based combination immunotherapy with oncogene inhibitors in patients with targetable tumors. Indeed, in-human clinical trials investigating such combinations are underway in RCC, breast cancer, and melanoma (Table 1).

## Immune Checkpoint Pathway Inhibitors

Immune checkpoint pathways – which under physiologic conditions prevent aberrantly activated T-cells from mediating autoimmunity – negatively regulate antitumor CTL function, rendering an “exhausted” T-cell phenotype. The CTLA-4/B7 and PD-1/PD-L1 pathways are areas of intense investigation. CTLA-4, a CD28 homolog, is upregulated upon T-cell activation and competes with CD28 for binding to APC ligands CD80 (B7.1) and CD86 (B7.2). Inhibitory CTLA-4-driven *signaling* in T-cells has historically been favored as the leading explanation for the therapeutic benefit of CTLA-4 blockade, reputedly resulting in TCR interference, attenuated IL-2 production, and cell cycle arrest (65, 66). Recent evidence, however, suggests a movement away from these signaling concepts toward a quantitative model of ligand competition, wherein the dominant function of CTLA-4 is control of CD28 access to shared ligands CD80/CD86 on APC/DCs (67). Intriguingly, the inhibitory function of CTLA-4 may be “domain-specific” – the extracellular, not cytoplasmic domain is sufficient to confer suppressive capacity (68), attenuating stimulatory CD28 signals via direct competition for APC ligands (67). Moreover, CTLA-4 inhibits CD28 co-stimulation by cell-extrinsic depletion of CD80 and CD86 on DCs via trans-endocytosis (69, 70); this downregulation can be abrogated either by CTLA-4 deficiency or blockade (71).

PD-1, a CD28/CTLA-4 homolog, is expressed on T-cells subjected to chronic antigen exposure (e.g., cancer, chronic infection, etc.). Analogous to exhausted T-cell phenotypes observed in murine models of chronic viral infection – which are partially reversed by PD-1 blockade (72) – TILs overexpressing PD-1 are thought to be functionally “exhausted” (73). Conventional wisdom holds that PD-1 binding to its ligands PD-L1/PD-L2 – expressed on myeloid cells, DCs, stromal cells, and tumor cells – provides inhibitory signals to T-cells (74). A more nuanced appreciation of PD-1 function, which better informs the therapeutic basis for PD-1 blockade in human cancer, has emerged recently. PD-1:PD-L1 engagement inhibits the TCR-induced “stop signal,” resulting in reduced T-cell:DC or T-cell:tumor contact; PD-1 blockade may reverse these effects, abrogate tolerance, and improve tumor targeting (75, 76). Moreover, PD-L1 induction on myeloid cells (including DC/APCs) in response to an inflammatory cytokine (e.g., IFN-γ) milieu can impair activation of tumor-specific T-cells (73, 77). Blockade of the PD-1:PD-L1 axis may counteract this adaptive resistance, restoring APC function, and enhancing T-cell-targeting of tumors; indeed, PD-L1 expression by infiltrating myeloid, rather than tumor cells was predictive of clinical response to PD-1 pathway blockade in a recently reported phase I study (78). Drawing on provocative evidence from chronic viral infection models, it now appears that PD-1 upregulation may not confer a terminally differentiated “exhausted” state, but rather perpetuates a functionally adapted and stable effector population capable of some degree of tumor control (79, 80). Collectively, these data may better explain rescue of T-cell function with PD-1 antagonism.

Monoclonal antibodies (mAb) targeting CTLA-4 and PD-1, therefore, have emerged as an attractive immunostimulatory strategy aimed at recovering T-cell function. In a seminal study, administration of anti-CTLA-4 mAb resulted in the rejection of pre-established tumors, as well as subsequent immunity to tumor rechallenge, in a murine model (81). The success of this, and other preclinical studies, precipitated the development, clinical testing, and subsequent FDA approval of anti-CTLA-4 mAb ipilimumab (82). More recently, preliminary evidence indicates that combination DC-based immunotherapy and CTLA-4 blockade may be synergistic in their benefit. In murine models of osteosarcoma and colorectal cancer, co-administration of anti-CTLA-4 mAb with either tumor lysate-loaded or immature DCs resulted in tumor growth inhibition, reduced metastasis, and enhanced survival (83, 84). In a phase I study in 16 advanced melanoma patients, co-administration of MART-1-pulsed DCs and anti-CTLA-4 mAb tremelimumab yielded more durable antitumor responses than with either agent alone (85).

The tumor *non-specific* mechanism of CTLA-4 blockade, however, manifests as dose-limiting toxicity in many patients (86). PD-1 blockade, conversely, is more tumor-*specific* and generates fewer adverse immune-related effects. Two FDA-approved anti-PD-1 mAb nivolumab and pembrolizumab have demonstrated tolerability and encouraging clinical responses in solid tumors (e.g., melanoma, non-small cell lung cancer, colorectal cancer, etc.) (87, 88) and hematologic malignancies (89). Approaches combining DC vaccines and PD-1 blockade are on the horizon – in a proof-of-principle study, anti-PD-1 mAb pidilizumab enhanced CD4^+^ and CD8^+^ T-cell responses following *ex vivo* stimulation with autologous myeloma-DC fusion vaccines (90). Trials testing pidilizumab in conjunction with DC vaccines in prostate cancer, RCC, and myeloma are underway (Table 1).

A related, but unintended, consequence of DC vaccination-induced Th1 immunity may be induction of PD-L1 expression on tumors. In our recent study, synergism between Th1 cytokines IFN-γ/TNF-α and trastuzumab strongly induced PD-L1 expression, in addition to class I upregulation, on HER2-overexpressing cells *in vitro*. While this phenomenon had minimal impact on DC1-sensitized HER2-specific CD8^+^ T-cell-mediated cytotoxicity of cancer cells – likely attributable to minimal PD-1 expression on activated CD8^+^ T-cells after limited *in vitro* DC1 sensitization (64) – these data justify exploration of a multidimensional therapeutic approach using DC vaccination, targeted therapies, and PD-1/PD-L1 blockade in patients with oncogene-driven tumors.

## Muting Immunosuppressive Phenotypes

In addition to co-inhibitory molecules, tumor-induced suppressive cellular networks (i.e., T_reg_ and MDSCs) also inhibit CTL function and mediate escape from immune surveillance. Three broad strategies to counteract T_reg_ and MDSCs are plausible. First, inhibiting T_reg_ (CD4^+^CD25^+^Foxp3^+^ T-cell) may augment DC efficacy. Antibodies targeting the IL-2 receptor α-chain CD25 (e.g., daclizumab, basiliximab) deplete T_reg_ and mediate tumor rejection in murine models. However, not only is this T_reg_ depletion effect transient but it also appears that these agents may paradoxically impair tumoricidal effector populations. In a phase I/II trial in 30 metastatic melanoma patients, addition of daclizumab to tumor antigen/KLH-pulsed DCs reduced circulating T_reg_, but undesirably suppressed tumor-specific CD25^+^ effectors. Progression-free survival was similar between daclizumab-treated vs. untreated patients (91). Denileukin diftitox – another CD25-targeting strategy – is a recombinant IL-2-diphtheria toxin conjugate demonstrating T_reg_ inhibition in RCC (92) and CEA-overexpressing malignancies (93). Paradoxically, however, denileukin induces a tolerogenic DC phenotype, promotes non-activated T_reg_ survival (94), and inhibits NK cells (95). A non-CD25-based alternative, 1-methyl-d-tryptophan – which inhibits indoleamine-2,3-dioxygenase (IDO) – may overcome these limitations, and is currently being investigated in combination DC-based immunotherapy trials (NCT01042535). In an alternative strategy, mAb targeting the anti-glucocorticoid-induced TNFR family-related receptor (GITR) – expressed highly in T_reg_ but not conventional T-cells – in conjunction with HER2/*neu*-expressing DC vaccines displayed potent antitumor immunity in a tolerogenic murine model (96). While promising, these T_reg_-targeting approaches must consider the risk of depleting T_reg_ systemically, which may generate irreversible autoimmunity.

Second, in light of evidence suggesting that MDSCs impair DC vaccine quality (97), concomitant targeting of these elements can be achieved by: (a) promoting MDSC differentiation into non-suppressive cells (e.g., all trans-retinoic acid, vitamin D3); (b) inhibiting myeloid cell development into MDSC (e.g., JAK2/STAT3 inhibitors, zolendronic acid); (c) depleting MDSC levels (e.g., sunitinib, gemcitabine, 5-FU); and (d) disabling MDSC function (e.g., cyclooxygenase-2 inhibitors, PDE-5 inhibitors, synthetic triterpenoids) (98, 99). Synthetic triterpenoids – such as bardoxolone methyl (CDDO-Me) – can inhibit JAK1/STAT3 signaling and reduce expansion of MDSCs (100). Dual treatment with a survivin-pulsed DC vaccine and CDDO-Me, compared with vaccination alone, delayed tumor progression and generated synergistic antigen-specific T-cell responses in EL-4 tumor-bearing mice (101).

Finally, DC vaccines can be designed to directly target immunosuppressive elements. Our group has demonstrated that LPS and IFN-γ-activated DC1 not only negate T_reg_ effects but also promote differentiation of these regulators into IFN-γ-secreting Th1 effectors (102). FoxP3 mRNA-transfected DC vaccines reduced intratumoral, but not systemic, FoxP3^+^ T_reg_ and bolstered TRP2-specific CTL responses following co-vaccination with TRP2-pulsed DCs in a murine melanoma model (103).

## Cytotoxic Chemotherapy

Increasing recognition of chemotherapy-induced immune effects have fueled the development of “chemoimmunotherapy” regimens that could be explored in conjunction with DC-based vaccination: (a) temozolomide or cyclophosphamide ± fludarabine reboots the immune system by eliminating immunosuppressive cells and creating an “immune recovery” cytokine (e.g., IL-7, IL-15) environment (44, 104); (b) Metronomically dosed cyclophosphamide depletes T_reg_/MDSCs, increases tumor cell permeability to CTL-derived cytolytic factors, and potentiates Th1 responses (44); (c) gemcitabine enhances tumor-associated antigen cross-presentation, while selectively mediating MDSC apoptosis (98, 105).

While the immune impact of such regimens is recognized, optimal sequencing of chemoimmunotherapy is yet to be definitively established. The potent immunogenicity of DC vaccines makes it an attractive strategy to boost antigen-specific immune responses in heavily pretreated patients – an interim analysis from our ongoing phase I trial investigating HER2-pulsed DC1 vaccination in HER2^pos^ breast cancer patients with residual disease following neoadjuvant chemotherapy/trastuzumab demonstrated robust anti-HER2 Th1 immunity 6 months post-vaccination (106). Intriguingly, administration of chemotherapy prior to immunization may even *bolster* antitumor immunity. In 35 non-Hodgkin’s lymphoma patients, pre-treatment with cyclophosphamide-containing regimens before tumor-derived idiotype-pulsed DC vaccination induced T-cell and humoral responses as well as generated durable tumor regression (107). Alternatively, DC pre-immunization may sensitize tumors to ensuing cytotoxic effects of chemotherapy. Following initial vaccination with tumor lysate/peptide-pulsed DCs, temozolomide-containing chemotherapy resulted in improved clinical responsivity and survival in glioblastoma patients (108). To confound matters, concomitant chemotherapy and DC vaccination may also be a feasible approach in particular tumor types – colon cancer patients *concurrently* receiving adjuvant oxaliplatin/capecitabine and KLH/CEA-pulsed DCs demonstrated CEA-specific T-cell responses (109). Trials attempting to elucidate the optimal dosing and timing of chemoimmunotherapy are underway (9).

## Radiotherapy

The traditional paradigm of viewing radiotherapy as merely cytoreductive has recently shifted to a more nuanced appreciation of its varied immunomodulatory effects (110). Such effects are exemplified in a recent study in which radiotherapy and dual checkpoint blockade (anti-PD1 plus anti-CTLA-4) demonstrated major tumor regression in metastatic melanoma patients via non-redundant immune mechanisms (111). The mechanistic rationale for addition of radiotherapy to DC-based interventions warrants discussion. Ionizing radiation (a) induces tumor cell apoptosis and necrosis secondary to vascular injury; phagocytosis and cross-presentation of apoptotic bodies by DCs primes tumor-specific T-cell responses if appropriate DC maturation signals are present (112); (b) upregulates expression of class I molecules (113), tumor-associated antigens (114, 115), death receptors, and NKG2D ligands on tumors, thereby enabling recognition and elimination of damaged cancer cells that have survived the cytocidal effects of radiotherapy (116); (c) induces generation of proinflammatory cytokines (TNF-α, IL-1β) or endogenous TLR agonists [HMGB1 (TLR4)], which activate DCs and potentiate antitumor inflammatory responses (117); (d) selectively inhibit immunosuppressive cellular (T_reg_) or soluble (TGF-β, VEGF) factors (118, 119), thereby enhancing DC functionality; (e) induce immune-mediated targeting of tumor stroma (120, 121), whereby antigen released after tumor irradiation may be presented by stromal cells for destruction by CTLs; and (f) inhibits distant *untreated* tumors – the so-called abscopal effect – via immune-mediated mechanisms (122).

This dynamic interplay between irradiated tumor, stromal cells, DCs/APCs, and effector/suppressive immune subsets has set the stage for clinical protocols combining radiotherapy with DC-based immunotherapy. Conformal radiotherapy followed by intratumoral injection of autologous immature DCs in refractory hepatocellular carcinoma patients generated partial responses and improvements in α-fetoprotein-specific immune responses in most patients (123). Autologous tumor lysate- or peptide-pulsed DCs were combined with intensity-modulated radiotherapy in 40 patients with advanced tumors; nearly two-thirds of patients receiving full-dose radiotherapy demonstrated objective responses (124). Trials investigating DC/radiotherapy protocols are ongoing in brain, breast, pancreatic, and esophageal cancer, as well as melanoma and sarcoma (125).

## TLR Agonists and/or Cytokines

Toll-like receptor agonists and cytokines – by virtue of their ability to regulate lymphocyte homeostasis and potentiate CTL function – are attractive adjuncts to DC-based vaccines. In preclinical studies, administration of TLR3 agonist poly(I:C) and peripheral vaccines resulted in robust Th1-polarized immunity and enhanced CTL activity. In a phase I/II clinical trial, co-administration of poly(I:C) with DC1 vaccines loaded with synthetic glioma-associated antigen epitopes demonstrated immunogenicity and improved progression-free survival in patients with CNS tumors (36). A phase I/II trial evaluating DC1 vaccines with tumor-selective chemokine modulation using poly(I:C) derivative rintatolimod, IFN-α, and COX-2 inhibitor celecoxib following resection of peritoneal surface malignancies is currently recruiting patients (NCT02151448). Other promising agents include TLR7/8 agonists (e.g., imiquimod, resiquimod), which stimulate TNF-α/IFN-α production by tumor-resident plasmacytoid DCs (126), and TLR9 agonists (e.g., CpG-containing oligodeoxynucleotides), which augment DC activation, enhance TME infiltration by effector T-cells, and inhibit T_reg_/MDSCs in preclinical models (127).

IL-2 is the most extensively studied systemic cytokine adjunct, with encouraging results in combinatorial approaches with DC-based vaccines in preclinical studies (128). Outcomes in the clinical setting are more equivocal – in a phase IB trial in 24 metastatic melanoma patients, treatment with autologous tumor lysate-pulsed DC vaccines and IL-2, albeit well tolerated and variably immunogenic, failed to induce meaningful objective responses (129). Other cytokine adjuncts hold promise – in the presence of IL-15, DCs are not only potent APCs but also express CD56 – an NK cell marker – which allow direct tumor cytotoxicity via elaboration of granzyme-B (130). Likewise, IL-7 potentiates DC activation in lymphoid tissue, and enhances TME infiltration of effector T-cells (131). Several combination DC-based immunotherapy trials utilizing these and other (e.g., IL-12, GM-CSF, IFN-γ, and pegylated-IFN-α) cytokines are currently underway (Table 1).

## Conclusion

Cancer immunotherapy – in particular, checkpoint inhibitors and genetically engineered T-cell receptor- or chimeric antigen receptor-directed T-cells – has emerged as a central approach in the “precision medicine” era. For DC-based immunotherapy to remain relevant in this rapidly changing clinical landscape, the paradigm must shift away from application of DC vaccines as monotherapy for solid tumors. Instead, a multifaceted approach incorporating versatile DC vaccine design and delivery, functionally synergistic targeted molecular and immune adjuncts/therapies, and rationally selected cytotoxic modalities (i.e., chemotherapy, radiotherapy) will yield the clinical outcomes that have remained elusive to date.

## Author Contributions

JD: conception and design, acquisition of data, writing/drafting manuscript, revising for important content, final approval of version to be published; agreement for accountability of published material; EB: acquisition of data, writing/drafting manuscript, revising for important content, final approval of version to be published; agreement for accountability of published material; JC: acquisition of data, writing/drafting manuscript, revising for important content, final approval of version to be published; agreement for accountability of published material; SX: acquisition of data, writing/drafting manuscript, revising for important content, final approval of version to be published; agreement for accountability of published material; RR: acquisition of data, writing/drafting manuscript, revising for important content, final approval of version to be published; agreement for accountability of published material; BC: conception and design, acquisition of data, writing/drafting manuscript, revising for important content, final approval of version to be published; agreement for accountability of published material.

## Conflict of Interest Statement

The authors declare that the research was conducted in the absence of any commercial or financial relationships that could be construed as a potential conflict of interest.

## Abbreviations

1-MT, 1-methyl-d-tryptophan; CTL, cytotoxic T-lymphocyte; CYC, cyclophosphamide; DC, dendritic cell; ID, intradermal; IDO, indoleamine-2,3-dioxygenase; IN, intranodal; GrB, granzyme-B; HER2, human epidermal growth factor receptor-2 (representative of receptor tyrosine kinase family); mAb, monoclonal antibody; MDSC, myeloid-derived suppressor cells; MHC/Ag, antigen presented in the context of MHC molecules; moDC, monocyte-derived DC; NK, natural killer cell; NKT, natural killer T-cell; PDGFR, platelet derived growth factor receptor; SC, subcutaneous; TCR, T-cell receptor; T_reg_, regulatory T-cell; VEGFR, vascular endothelial growth factor receptor.
